# The occurrence of musculoskeletal complaints among professional musicians: a systematic review

**DOI:** 10.1007/s00420-015-1090-6

**Published:** 2015-11-12

**Authors:** Laura M. Kok, Bionka M. A. Huisstede, Veronique M. A. Voorn, Jan W. Schoones, Rob G. H. H. Nelissen

**Affiliations:** Department of Orthopedics, Leiden University Medical Center, P.O. Box 9600, 2300 RC Leiden, The Netherlands; Department of Orthopedics, Spaarne Hospital, Hoofddorp, The Netherlands; Department of Rehabilitation, Nursing Science and Sports, University Medical Center Utrecht, Utrecht, The Netherlands; Department of Medical Decision Making, Leiden University Medical Center, Leiden, The Netherlands; Walaeus Library, Leiden University Medical Center, Leiden, The Netherlands

**Keywords:** Occupational, Epidemiology, Arts, Music, Musician, PRMD

## Abstract

**Purpose:**

This study gives a systematic overview of the literature on the occurrence of musculoskeletal complaints in professional instrumental musicians.

**Methods:**

A systematic review. Nine literature databases were searched without time limits on June 25, 2015, also the complete index of the journal Medical Problems of Performing Artists (MPPA) until June 2015 (30;2) was searched, and citation tracking and reference checking of the selected articles were performed. The search consisted of the combination of three groups of keywords: musician (e.g., musician, violin, music student, instrument player) AND musculoskeletal (e.g., musculoskeletal, tendon, shoulder, arthritis) AND epidemiology (e.g., prevalence, incidence, occurrence).

**Results:**

The initial literature search strategy resulted in 1258 potentially relevant articles. Finally, 21 articles describing 5424 musicians were included in this review. Point prevalences of musculoskeletal complaints in professional musicians range between 9 and 68 %; 12-month prevalences range between 41 and 93 %; and lifetime prevalences range between 62 and 93 %. Ten out of 12 studies show a higher prevalence of musculoskeletal complaints among women. Brass instrumentalists are reported to have the lowest prevalence rates of musculoskeletal complaints. The neck and shoulders are the anatomic areas most affected; the elbows are least affected. Although some information is reported concerning age, the high risk of bias in and between these studies makes it impossible to present reliable statements with respect to this.

**Conclusion:**

Musculoskeletal symptoms are highly prevalent among musicians, especially among women instrumentalists. Future research concerning the epidemiology of musculoskeletal complaints among musicians should focus on associated risk factors and follow the current guidelines to optimize scientific quality.

## Introduction

‘There is no exercise, though never so healthful and innocent, but what may produce great disorders, if it is used with intemperance,’ are the words of Bernardino Ramazzini, who was in 1713, the first to describe an overview of occupational diseases of musicians (Sataloff et al. 2010; Bejjani et al. 1996). Only at the end of the nineteenth century, a number of physicians turned their interest to some specific musicians’ complaints like musicians’ cramp. Tenotomies of the finger flexors were performed in order to improve finger independency among pianists (Sataloff et al. 2010). However, real interest in the health and well-being of musicians by medical practitioners, researchers and music professionals was developed since the 1980s. This was reflected in a growing number of publications, journals, conferences, and organizations focused on the health of the performing artists (Sataloff et al. 2010; Bejjani et al. 1996). Nowadays, the level of knowledge on this topic and the necessary specialized healthcare is still in a developmental stage when compared to, for instance, sports medicine, and thus room for improvement remains.

Musculoskeletal complaints are one of the main medical problems among musicians (Guptill and Golem 2008; Hoppmann and Reid 1995; Heinan 2008). These complaints have considerable physical, psychological, social and financial impact on musicians (Spahn et al. 2001; Zaza et al. 1998). Impaired level of functioning at both work and daily activities at home due to these musculoskeletal complaints is reported in the majority, and sleep disturbances related to these complaints are reported in half of the professional musicians (Paarup et al. 2011; Kaneko et al. 2005). Most professional musicians will suffer from musculoskeletal complaints during their life; some of them will stop playing their instrument due to these complaints (Kaufman-Cohen and Ratzon 2011; Parry 2003; Kaneko et al. 2005).

Zaza published in 1998 a systematic review of incidence and prevalence of playing-related musculoskeletal complaints (Zaza 1998). In this study, 18 cross-sectional and cohort studies published between 1980 and 1996 were reviewed. Due to different definitions of musculoskeletal complaints, the point prevalence of the playing-related musculoskeletal disorders varied between 39 and 87 %. A development since this review is the introduction of the term playing-related musculoskeletal disorder (PRMD) (Zaza et al. 1998), which aims to exclude minor irrelevant musculoskeletal symptoms experienced by musicians. Zaza defined PRMDs as personal, chronic and disabling health problems that affect the whole person, physically, emotionally, occupationally and socially (Zaza et al. 1998). However, the term PRMD is used in the literature of performing arts medicine without strictly following this definition. Recently, another review was published concerning pain prevalence in musicians (Silva et al. 2015). In this review, heterogenic studies are compared, with no distinction between professional and amateur musicians, impeding extrapolation of these results.

An up-to-date critical systematic review of the literature to assess prevalence rates of musculoskeletal complaints among musicians will indicate the extent of the problem, and a critical appraisal of the used prevalence rates and definitions of studied complaints will give an overview of the current science of musculoskeletal problems in musicians. Furthermore, subgroups with a higher prevalence can be identified. This may be helpful in the prevention of complaints due to the possibility to target prevention and interventions at these high risk groups.

Therefore the objective of this systematic review is to give an overview of the prevalence of musculoskeletal complaints among professional instrumental musicians and to evaluate groups and localizations at risk.

## Materials and methods

### Search strategy

A literature search was performed on June 25, 2015, using the following databases without time and language restrictions: PubMed, Embase, Web of Science, Cochrane, Cumulative Index to Nursing and Allied Health Literature (CINAHL), Academic Search Premier, PsycINFO, ScienceDirect and Lippincott Williams & Wilkins (LWW). The search consisted of the combination of three groups of keywords: musician (e.g., musician, violin, music student, instrument player) AND musculoskeletal (e.g., musculoskeletal, tendon, shoulder, arthritis) AND epidemiology (e.g., prevalence, incidence, occurrence). The complete search strategy is presented in “Appendix A”. Moreover, the complete index of the journal Medical Problems of Performing Artists (MPPA) until June 2015 (30;2) was searched manually, and citation tracking and reference checking of the selected articles was performed.

### Inclusion criteria

Articles were included if they fulfilled all of the following criteria: (1) the study had a cross-sectional, case–control or cohort design; (2) the study population consisted of adult (aged 18 or older) professional instrumental musicians and/or music academy students; the definition of professional was dependent on the definition of the original article. (3) The outcome measure reported was a clearly described prevalence rate of musculoskeletal complaints of the complete body or half of the body (at least upper extremities, back and neck) of musicians; (4) the article was published in a peer-reviewed scientific journal. If a subset of the total number of subjects included in a study met our inclusion criteria, the study was included only if the outcomes of the subset were assessed and reported independently.

### Exclusion criteria

Studies with subjects aged 17 or younger were excluded. In case of unclear age limits, an indistinct description of the prevalence rate or questions concerning the professionalism of the study subjects, the authors were sent an e-mail. In case of non-response, the study was excluded. Case series that included less than 50 subjects were excluded. Also studies reporting a prevalence of musculoskeletal complaints measured within a population visiting a healthcare professional were excluded. In case of a mixed study population, in which only a part of the study subjects met the inclusion criteria, authors were e-mailed and asked for split results. In case of a non-responding author or the inability of the author to present the relevant information, the study was excluded.

### Study selection

Two reviewers (L.M.K., V.M.A.V.) independently performed the screening of title, abstract and full-text articles respectively, on eligibility. Disagreements in the selection process were resolved by consensus. When no consensus was reached, a third reviewer (B.M.A.H.) was consulted. In case of incomplete information in potentially relevant studies, the author was contacted by e-mail twice.

### Data extraction

Two reviewers (L.M.K., V.M.A.V.) independently extracted the data from the included articles. General manuscript information (authors, title, year and journal) was collected. Information on the study population, sample size and response rate was listed. The prevalence rates of musculoskeletal complaints and specifications of these prevalence rates for differences in age, gender, occupation, localization and type of instrument were made. We also recorded whether the musculoskeletal complaints were playing related (yes/no). Disagreement between the reviewers was resolved by consensus.

### Assessment of methodological quality

The methodological quality assessment was performed using a scoring system developed by Loney et al. (1998) and Shamliyan et al. (2010). This scoring system is specifically designed for studies reporting incidence and prevalence rates and consists of an eight-point checklist. Table 1 shows the quality criteria in eight categories: design and method; sampling; sample size; measurement criteria; bias; response and non-responders; outcomes; setting. A score ranging between zero (lowest score) and eight (highest score) indicates the quality of the included study (Table 1). Two independent reviewers (L.M.K., V.M.A.V.) assessed the quality of the studies. Disagreements were resolved by consensus. When no consensus was found, a third reviewer (B.M.A.H.) was consulted if the disagreement persisted.

**Table 1:** Methodological quality scoring system

	Study-specific requirements
1. Are the study design and sampling method appropriate for the research question?	Is it an observational study? And is there an adequate sample of the total population studied in the research question?
2. Is the sampling frame appropriate?	Is the ‘list for study recruitment’ from which subjects are selected (sampling frame) appropriate? (no under- or overrepresentation of the problem in the subpopulation?)
3. Is the sample size adequate?	An adequate sample size calculation in this study and/or *n* > 100
4. Are objective suitable and standard criteria used for measurement of the health outcome?	Are validated questionnaires used?
5. Is the health outcome measured in an unbiased fashion?	Is there a possible bias in the interpretation of the results?
6. Is the response rate adequate? Are the refusers described?	>66.6 % response rate and dropouts described and compared with the study population
7. Are the estimates of prevalence or incidence given with confidence intervals and in detail by subgroup if appropriate?	
8. Are the study subjects and the setting described in detail and similar to those of interest to you?	Are the sociodemographic characteristics adequately described?
Total	0–8 Points Scoring system: 0–4 points = low; 5–8 points = high

### Pooling of data

The aim was to pool the data if there would be sufficient homogeneity between the included studies.

## Results

### Study selection

**Fig. 1:**
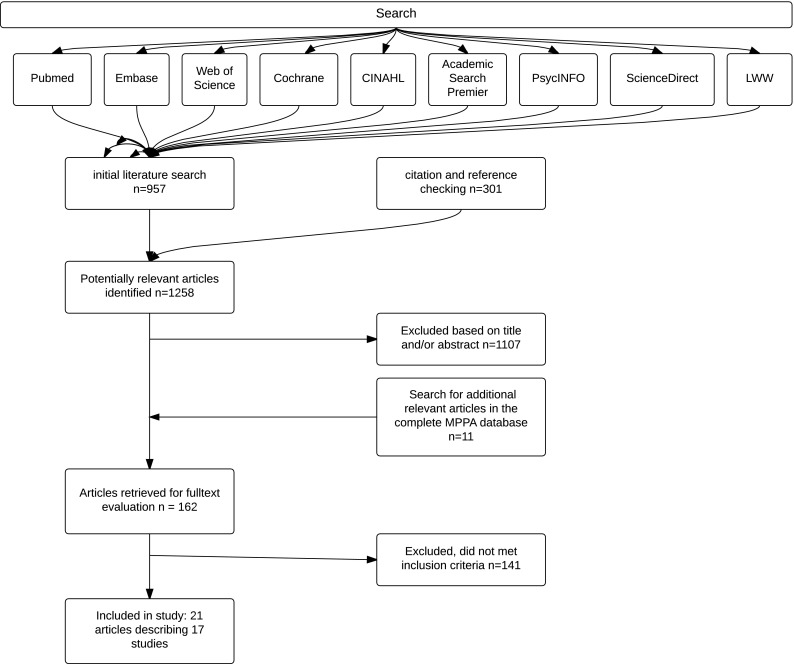
Systematic literature review process

The initial literature search strategy resulted in 957 potentially relevant articles. Another 301 articles were identified after citation tracking and by checking the references of the selected articles. After the screening of title and abstract, 162 articles were considered eligible for inclusion and the full text was screened. Searching the MPPA database resulted in another 11 articles selected for full-text assessment. Finally, 21 articles, describing 17 studies and 5424 professional instrumental musicians, met our inclusion criteria and were included. Three study populations were reported on more than one article (Kok et al. 2013a, b; Fishbein et al. 1988; Middlestadt and Fishbein 1988, 1989; Ackermann et al. 2012; Kenny and Ackermann 2015); results of these studies were pooled and presented as a single study. A flowchart of the inclusion and exclusion process is presented in Fig. 1.

Articles without a clear description of age of the study population, (Fjellman-Wiklund and Chesky 2006; Kreutz et al. 2008; Hagglund and Jacobs 1996; Larsson et al. 1993; Miller et al. 2002; de Sousa et al. 2014) and articles lacking a clearly described type of prevalence rate (Mishra et al. 2013; Brandfonbrener 1997; Nemoto and Arino 2007; Mehrparvar et al. 2012; Hagglund and Jacobs 1996; Marques et al. 2003; Fjellman-Wiklund and Chesky 2006; Eller et al. 1992; Caldron et al. 1986; Wood 2014; Lopez and Farias 2013) were excluded. Also, studies with a mixed under aged population (Barton et al. 2008; Zetterberg et al. 1998; Caldron et al. 1986; Shields and Dockrell 2000; Wood 2014) or mixed occupation (Arnason et al. 2014; Wristen and Fountain 2013) (e.g., partly conductors or singers and not primary instrumental musicians) in which the authors were not able to present split data were excluded. Many articles derived from the UNT-MHS database (Chesky 2000) had to be excluded after e-mail contact with the main author due to the lack of a professional study population.

### Characteristics of the included studies

Table 3 gives an overview of the included studies. All included studies had a cross-sectional design. In fourteen articles symphony orchestra musicians were studied, in four articles music academy students, and in three other articles a mixed population of professional musicians and music academy students were studied. The included studies showed a variety of gender distributions, 26–79 % of the in the separate studies included participants were male. The studies were performed on a variety of continents: seven European, one Asian, five North American, two South American and two Oceanic studies were included. Response rates to questionnaires varied between 26 and 99 %; one study did not report a response rate (Arnason et al. 2014); and the number of participants of each separate study ranged between 59 and 2212.

### Assessment of methodological quality

The results of the methodological quality assessment are presented in Table 2. Only two studies reported an adequate response rate (over 66.6 %) and an adequate description of the non-responders (Paarup et al. 2011; Engquist et al. 2004). Three studies used a validated outcome measure (Leaver et al. 2011; Kaufman-Cohen and Ratzon 2011; Engquist et al. 2004; Fotiadis et al. 2013), compared to 14 studies which used non-validated outcome measures or non-validated modifications of existing questionnaires. Overall, the quality of the included studies was variable; however, many studies of low scientific quality were excluded from this review due to the strict exclusion criteria for reporting outcomes (Table 3).

**Table 2:** Methodological quality score of the included articles

	1. Design and sampling method	2. Sampling frame	3. Sample size	4. Objective, suitable, standard criteria	5. Measured unbiased	6. Response rate adequate and describing non-participants	7. Confidence intervals	8. Interest for this study	Total score	Quality of the study
Leaver et al. (2011)	1	1	1	1	1	0	0	1	6	High
Paarup et al. (2011)	1	1	1	0	1	1	1	1	7	High
Kaufman-Cohen et al. (2011)	1	1	0	1	1	0	0	1	5	High
Zaza and Farewell (1997)	1	1	1	0	1	0	0	1	5	High
Abreu-Ramos and Micheo (2007)	1	1	0	0	1	0	0	1	4	Low
Kaneko et al. (2005)	1	1	1	0	1	0	0	1	5	High
Engquist et al. (2004)	1	1	1	1	1	1	0	1	7	High
Davies and Mangion (2002)	1	1	1	0	1	0	0	1	5	High
Roach et al. (1994)	1	0	0	0	1	0	0	1	3	Low
O’Neill et al. (2001)	1	1	1	0	1	0	0	1	5	High
Kok et al. (2013a, b)	1	1	1	0	1	0	0	1	5	High
Fishbein et al. (1988); Middlestadt and Fishbein (1988, 1989)	1	1	1	0	1	0	0	1	5	High
Ackermann et al. (2012), Kenny and Ackermann (2015)	1	1	1	0	1	0	0	1	5	High
Arnason et al. (2014)	1	1	0	0	0	0	0	0	2	Low
Steinmetz et al. (2015)	1	1	1	0	1	0	0	1	5	High
Chimenti et al. (2013)	1	1	1	0	1	0	0	0	4	Low
Fotiadis et al. (2013)	1	1	1	1	1	0	0	1	6	High

**Table 3:** Characteristics of the included studies

	Response rate (%)	Number participants (*n*)	Age	Gender (% male)	Instruments (%)	Job characteristics	Definition or description of ‘musculoskeletal complaints’	Localization of complaints
Leaver et al. (2011)	51	243	Mean 44 (range 23–64)	56	String 62 % Woodwind 15 % Brass 16 % Other 17 %	British symphony orchestra musicians	‘The questions on musculoskeletal symptoms were based on the standardized Nordic questionnaire and concerned pain’ ‘disabling pain was defined as pain in the past 12 months present for at least a month and which prevented attendance at work for at least 1 day’	Low back, neck, upper extremities
Paarup et al. (2011)	78	441	Mean men 48 (CI 46–50) Mean women 39 (CI 37–43)	61	String 47 % Woodwind 14 % Brass 12 % Other 4 %	Danish symphony orchestra musicians	‘…adapted from the Nordic Musculoskeletal Questionnaire. The symptoms were measured as presence of trouble (ache, pain, or discomfort)’ ‘As in the DASH questionnaire four questions were asked to assess if the symptoms had led to difficulties in playing, but the time span of interest was extended to 12 months’	Neck, back, upper extremities
Kaufman-Cohen and Ratzon (2011)	66	59	Mean 43 (range 26–66 SD 11)	49	String 66 % Wind 34 %	Israelian symphony orchestra musicians	‘The presence of pain’	Upper extremities
Zaza and Farewell (1997)	67	281	Mean 31	45	String 33 % Other 67 %	USA classically trained musicians and university music students	‘Playing-related musculoskeletal problem (i.e. any pain, weakness, numbness, tingling, or other symptoms that interfere with your ability to play your instrument at the level you are accustomed to)’	Not specified
Abreu-Ramos and Micheo (2007)	90	75	Mean 38 ± 10 (Range 22–61)	79	String 43 % Woodwind 15 % Brass 17 % Percussion 4 % Other 1 %	Puerto Rico symphony orchestra musicians	‘Questions related to musculoskeletal problems, including pain, allodynia, and dysesthesias (expressed as burning, electrical sensation, ‘pins and needles’, tingling, numbness), weakness, cramps and involuntary movements’	Neck, back, upper extremities, mouth
Kaneko et al. (2005)	56	241	Mean 32 (range 18–73, SD 11)	70	String 61 % Woodwind 17 % Brass 12 % Percussion 8 % Conductor 2 %	Brazilian symphony orchestra musicians	Pain. ‘The McGill pain questionnaire was used to specify subjective pain experience using sensory, affective and evaluative word descriptors, and a body diagram was used to locate the pain’	Head, neck, back, upper extremities
Engquist et al. (2004)	43	103	Mean 40 (20–61)	61	String 56 % Wind 36 % Other 5 %	Swedish orchestra musicians	Extension of the Standardized Nordic Questionnaire	Total body
Davies and Mangion (2002)	45	240	Mean 37 (SD 11)	56	String 42 % Woodwind 18 % Brass 16 % Percussion 7 % Keyboard 12 % Guitar 5 %	Australian classical and non-classical instrumental musicians	‘Playing-related pain and/or symptoms (pins and needles, swelling, muscle weakness or loss of control)’	Not specified
Roach et al. (1994)	99	90	Mean 23	54		USA music academy instrumentalists	‘Subjects were asked to report joint pain for any site at which they had experienced pain for at least 2 days during the preceding 4 weeks’	Neck, upper back, upper extremities
O’Neill et al. (2001)	50	103	Mean 36 (range 18–66)	49	String 34 % Trumpet 9 % Piano 8 % Percussion 8 %	Instrumentalists from orchestras, a music academy and privately studying musicians in Canada	‘Respondents reported that they had experienced pain as a result of playing their instrument at least once in the course of their musical studies’ ‘Chronic injury, that is, pain lasting for longer than 3 months’	Not specified
Kok et al. (2013a, b)	26	83	Mean 22 (SD 2) (>18)	26	String 39 % Woodwind 33 % Brass 8 % Percussion and keyboard: 20 %	Dutch music academy students	‘Questions on each of these body regions started by asking about complaints of—the specific body region—during the last 12 months’	Total body
Fishbein et al. (1988); Middlestadt and Fishbein (1988, 1989)	55	2212	Mean 42	64	String 62 % Woodwind 16 % Brass 16 % Percussion 4 % Keyboard: 1 % Other: 1 %	USA orchestra instrumentalists	‘Thus musicians were asked to circle the number of all those problems they had experienced’ ‘… of ICSOM musicians at one particular point in time. Thus, the data reflect prevalence, but not incidence’	Total body
Ackermann et al. (2012), Kenny and Ackermann (2015)	70	377	Mean 42 (SD 10)	49	Strings 63 % Woodwinds 18 % Brass 15 % Percussion 3 % Other 1 %	Australian symphony orchestra musicians	‘Performance-related musculoskeletal disorders were defined as ‘any pain, weakness, numbness, tingling or other physical symptoms that interfere with your ability to play your instrument at the level to which you are accustomed. This definition does not include mild transient aches or pains’	Total body
Arnason et al. (2014)	?	60 (74 including vocalists)^a^	Mean 22 (SD 4)	57	Strings: 32 % Woodwinds and brass: 23 % Keyboard: 23 % Percussion: 4 % Vocalists: 19 *%* ^a^	Icelandic music school students	‘Both the cumulative and current prevalence, as well as the severity of PRMD among musicians’ Adapted and translated formerly used questionnaire	Total body
Steinmetz et al. (2015)	57	408	Mean 44 (SD 10)	58	Strings: 56 % Woodwinds: 15 % Brass: 14 % Percussion: 3 % Other: 3 % Missing: 10 %	German orchestra musicians	‘Regarding playing-related musculoskeletal pain, participants were asked whether they had experienced current or past pain in several body regions’	Total body
Chimenti et al. (2013)	26	261	Range 22–75; 78 % between 30 and 59	47	Strings: 66 % Woodwinds: 18 % Brass: 14 % Other: 3 %	Professional orchestra musicians of the international conference of symphony and opera musicians	‘Playing-related symptoms, including but not limited to: pain, weakness, stiffness, swelling, numbness, and/or decreased coordination associated with playing’	Total body
Fotiadis et al. (2013)	60	147	Mean 39 (SD?)	66	Strings: 63 % Woodwinds: 17 % Brass: 14 % Percussion: 5 % Other: 1 %	Greek professional orchestra musicians	Standardized Nordic Questionnaire	Total body

^a^Vocalists excluded in the reviewed prevalence rates

### Prevalence rates

A uniform definition of musculoskeletal complaints in the included studies was lacking; some authors used the definition of playing-related musculoskeletal complaints by Zaza et al. (1998) and Ackermann et al. (2012) (‘any pain, weakness, numbness, tingling, or other symptoms that interfere with your ability to play your instrument at the level you are accustomed to’), whereas others (Engquist et al. 2004; Leaver et al. 2011; Paarup et al. 2011; Kaufman-Cohen and Ratzon 2011) used the questions based on the standardized Nordic Questionnaire (Leaver et al. 2011; Kuorinka et al. 1987; Fotiadis et al. 2013; Paarup et al. 2011; Engquist et al. 2004). In other studies, different descriptions such as ‘(joint-) pain,’ or ‘trouble’ to describe the complaints were used (Kaneko et al. 2005; Kok et al. 2013a; Fishbein et al. 1988; Abreu-Ramos and Micheo 2007; Poolman et al. 2009).

Among the included studies, there was heterogeneity in the type of prevalence rates. Point prevalence, 12-month prevalence and life-time prevalence were most frequently reported. However, also 4-week prevalence, ‘chronic’ prevalence (with different definitions in the two reporting studies), and 3-month and 2-year prevalence were reported.

Two studies concerned all musculoskeletal complaints (without making a difference between playing-related or other complaints), whereas 12 studies measured only playing-related complaints. Three studies reported both playing-related and all musculoskeletal complaints. This variety of definitions of ‘musculoskeletal complaints,’ the heterogeneity of the reported prevalence types and the variability within study populations made it impossible to pool the data in this review.

### Prevalence rates

Reported point prevalence rates of musculoskeletal complaints, presented in Table 4, varied from 57 to 68 % for all musculoskeletal complaints, and from 9 to 68 % for playing-related complaints. Non-playing-related 12-month prevalence ranged between 86 and 89 %, and playing-related 12-month prevalence ranged between 41 and 93 %. Playing-related lifetime prevalence ranged between 62 and 93 %. No study reported non-playing-related lifetime prevalence.

**Table 4:** Total prevalence rates of musculoskeletal symptoms among professional musicians

	Point prevalence, not playing related (%)	Point prevalence, playing related (%)	12-month prevalence, not playing related (%)	12-month prevalence, playing related (%)	Life-time prevalence, not playing related (%)	Life-time prevalence, playing related (%)	Other prevalence (%)
Leaver et al. (2011)			86	41			71^a^
Paarup et al. (2011)			88	73			
Kaufman-Cohen and Ratzon (2011)				83			
Zaza and Farewell (1997)		39				70	
Abreu-Ramos and Micheo (2007)						81	
Kaneko et al. (2005)	68/57^b^						
Engquist et al. (2004)	61			52			47^c^
Davies and Mangion (2002)		50				93	
Roach et al. (1994)							67^d^
O’Neill et al. (2001)						90	58^e^ 36^f^
Kok et al. (2013a, b)	63		89				
Fishbein et al. (1988), Middlestadt and Fishbein (1988, 1989)		68					
Ackermann et al. (2012), Kenny and Ackermann (2015)		50				84	
Arnason et al. (2014)						62	
Steinmetz et al. (2015)		9				90	63^g^
Chimenti et al. (2013)				93			
Fotiadis et al. (2013)						82	

^a^Four-week prevalence, not playing related

^b^Differences in reported prevalence rates in text and Tables

^c^Chronic, not playing related (chronic defined as often, or all the time, in contrast to no, never, once or twice, or sometimes during the past 12 months)

^d^Four-week prevalence of pain at least 2 days present

^e^Two-year prevalence, playing related

^f^Pain lasting longer than 3 months (chronic injury)

^g^Pain within the last 3 months, playing related

### Gender

Ten out of 12 studies comparing the gender of the professional musicians showed a higher prevalence of musculoskeletal complaints among women. One study (Kaufman-Cohen and Ratzon 2011) only stated ‘no significant difference’ without presenting the data, and another study (Davies and Mangion 2002) reported a higher prevalence among female compared with male strings players, but a lower prevalence among females playing another instrument. However, no exact data were given in this study. Table 5 shows the results of the gender-specific prevalence rates.

**Table 5:** Prevalence rates of musculoskeletal complaints among professional musicians; gender-specific results

	Type of prevalence	Specification body part	Men (%)	Women (%)	OR (SD) women compared to men	Other information on sex differences in the manuscript
Leaver et al. (2011)	12-month prevalence, not playing related	Low back	47	56	1.4 (0.9–2.4)	
		Neck	48	65	2.0 (1.2–3.3)	
		Shoulders	42	62	2.2 (1.3–3.8)	
		Elbows	24	17	0.6 (0.3–1.2)	
		Wrists	29	37	1.4 (0.8–2.5)	
Paarup et al. (2011)	12-month prevalence, not playing related	Total	83	97		
		Neck			2.9 (1.9–4.6)	
		Upper back			2.8 (2.1–3.8)	
		Lower back			1.3 (0.8–2.4)	
		Left shoulder			2.4 (1.6–3.7)	
		Right shoulder			3.2 (1.8–5.6)	
		Left elbow			3.5 (1.2–10.1)	
		Right elbow			1.7 (0.8–3.6)	
		Left hand and wrist			3.3 (1.6–7.2)	
		Right hand and wrist			2.1 (1.5–3.0)	
Kaufman-Cohen and Ratzon (2011)	12-month prevalence, playing related	Upper extremities				‘No significant differences’
Zaza and Farewell (1997)	Point prevalence, playing related	Not specified			2.8 (1.1–7.5)	
Abreu-Ramos and Micheo (2007)	Lifetime prevalence, playing related	Neck, back, upper extremities, mouth	80	88		
Kaneko et al. (2005)	Point prevalence, not playing related	Head, neck, back, upper extremities				*p* < 0.001
Engquist et al. (2004)	Point prevalence, not playing related	Neck, back, shoulders	26	37		
Davies and Mangion (2002)		Not specified				‘Female string players were more affected in the previous 12 months than male strings players. Related to his last result is the finding that for the previous year, men were significantly more affected by pain/symptoms than women, unless the women were string players’
Roach et al. (1994)	4-week prevalence, not playing related	Neck, upper back, upper extremities	61	73	1.7 (0.7–4.2)	
O’Neill et al. (2001)	2-year prevalence, playing related	Not specified	56	60		
Kok et al. (2013a, b)		Total body				No information presented
Fishbein et al. (1988); Middlestadt and Fishbein (1988); Middlestadt and Fishbein (1989)	Point prevalence, playing related	Total body	52	70		(*p* < 0.05)
Steinmetz et al. (2015)	Lifetime prevalence, playing related	Total body	88	92		

### Occupation

There were no studies that compared prevalence rates of musculoskeletal complaints between different occupational groups (e.g., orchestral musicians, music teachers, music academy students).

Kok et al. (2013a, b) reported a point prevalence of 63 % of musculoskeletal complaints among music academy students. The latter was in concordance with Kaneko et al. (2005) and Engquist et al. (2004) who reported prevalence rates of 68 and 61 % respectively, in orchestra musicians. Also the 12-month prevalence of 89 % among music academy students in the study of Kok et al. was comparable to the prevalence rates of orchestra musicians of Leaver et al. (2011) and Paarup et al. (2011), 86 and 88 % respectively.

No information was presented in the included articles concerning prevalence rates between different occupations among professional musicians; e.g., teachers, chamber musicians, soloists and orchestra musicians.

### Instrument

In addition to the above-mentioned heterogeneity in the definition of measured complaints and the type of prevalence reported in each study, heterogeneity in the grouping of instrumentalists and the presentation of differences between these instrument groups were presented in the included studies. Some authors reported no total prevalence rate split for instrument groups, only body-area-specific prevalence rate split for instrument groups (Leaver et al. 2011; Paarup et al. 2011; Roach et al. 1994). As there is a possibility to have multiple complaints, these numbers could not be summed up. An overview of the reported prevalence rates for each instrument group is presented in Table 6. Overall, no specific instrument group had an evidently higher prevalence rate of musculoskeletal complaints. However, brass instrumentalists were reported to have the lowest prevalence rates of musculoskeletal complaints (Arnason et al. 2014; Leaver et al. 2011; Paarup et al. 2011; Abreu-Ramos and Micheo 2007; Kaneko et al. 2005; Roach et al. 1994; Kok et al. 2013a; Fishbein et al. 1988; Ackermann et al. 2012; Steinmetz et al. 2015).

**Table 6:** Prevalence rates of musculoskeletal complaints among professional musicians; instrument specific results

	Type of prevalence	Specification body part	Strings, prevalence	OR strings^a^	Prevalence woodwinds	OR woodwinds^a^	Prevalence brass	OR brass^a^	Other^a^
Leaver et al. (2011)	12-month prevalence, not playing related	Back		1.00		0.8 (0.4–1.7)		0.5 (0.2–1.0)	1.1^b^ (0.4–3.2)
		Neck		1.00		2.5 (1.1–6.0)		1.0 (0.4–2.1)	1.4^b^ (0.5–4.2)
		Shoulders		1.00		1.1 (0.5–2.5)		0.7 (0.3–1.7)	0.5^b^ (0.2–1.6)
		Elbows		1.00		0.6 (0.2–1.7)		0.4 (0.1–1.2)	1.0^b^ (0.3–3.2)
		Wrists/hands		1.00		2.9 (1.3–6.7)		0.4 (0.2–1.2)	2.6^b^ (0.8–7.7)
Paarup et al. (2011)	12-month prevalence not playing related; OR compared with high strings	Neck		High: 1.0 Low: 1.0 (0.6–1.6)		0.5 (0.3–0.7)		0.8 (0.3–2.1)	0.6^b^ (0.3–1.6)
		Upper back		High: 1.0 Low: 1.4 (0.6–3.0)		1.0 (0.5–2.0)		0.9 (0.4–1.9)	1.5^b^ (0.8–2.9)
		Lower back		High: 1.0 Low: 0.7 (0.4–1.5)		0.5 (0.3–0.8)		0.8 (0.3–2.2)	0.8^b^ (0.2–3.2)
		Left shoulder		High: 1.0 Low: 0.6 (0.3–1.1)		0.5 (0.3–0.8)		1.2 (0.6–2.4)	0.3^b^ (0.1–0.8)
		Right shoulder		High: 1.0 Low: 1.7 (0.7–3.9)		0.8 (0.3–2.1)		1.3 (0.6–2.7)	0.8^b^ (0.1–5.2)
		Left elbow		High: 1.0 Low: 1.5 (0.6–3.9)		0.4 (0.1–1.9)		1.7 (0.9–3.4)	4.7^b^ (1.2–18.4)
		Right elbow		High: 1.0 Low: 1.1 (0.5–2.5)		1.0 (0.4–2.6)		0.6 (0.2–2.1)	1.2^b^ (0.4–3.4)
		Left hand and wrist		High: 1.0 Low: 1.3 (0.7–2.6)		0.5 (0.2–1.2)		0.8 (0.4–1.8)	1.1^b^ (0.2–6.7)
		Right hand and wrist		High: 1.0 Low: 1.8 (0.8–3.9)		1.2 (0.5–2.7)		0.4 (0.2–0.8)	1.8^b^ (0.4–7.5)
Kaufman-Cohen and Ratzon (2011)	12-month prevalence, playing related	Upper extremities							‘No statistically significant difference between string and wind musicians’
Zaza and Farewell (1997)	Point prevalence, playing related	Not specified							Strings compared to keyboard OR 4.7 (CI 1.5–14.5)
Abreu-Ramos and Micheo (2007)	Lifetime prevalence, playing related	Neck, back, upper extremities, mouth	High 78 % Low 93 %		82 %		69 %		100 %^b^
Kaneko et al. (2005)	Point prevalence, not playing related	Neck, back, upper extremities, mouth	69 %		65 %		55 %		55 %^b^ All differences *p* > 0.05
Engquist et al. (2004)	12-month prevalence, playing related	Head, neck, back, upper extremities	39 %	OR 2.0 (0.7–5.2) compared with non-string (adjusted for gender and age)					Non-strings: 23 %
Davies and Mangion (2002)		Neck, back, shoulders							‘String players were significantly more likely to have frequent and severe pain/symptoms over the playing lifetime’
Roach et al. (1994)	4-week prevalence, not playing related	Not specified		3.7 (1.4–9.2)^i^				0.5 (0.1–1.8)^h^	1.4 (0.8–2.6)^c^ 1.7 (0.6–5.1)^d^ 1.4 (0.5–3.7)^b^
		Upper back		6.3 (2.6–15.2)^i^				0.6 (0.1–2.8)^h^	0.8 (0.1–3.1)^d^ 1.6 (0.8–3.1)^b^ 0.5 (0.1–2.1)^c^
		Shoulder		6.5 (2.7–15.6)^i^				0.1 (0.0–2.1)^h^	0.7 (0.1–3.2)^d^ 1.6 (0.8–3.0)^b^ 1.2 (0.4–3.9)^c^
		Elbow		4.4 (0.9–20.5)^i^				0.9 (1.1–16.7)^h^	13.8 (4.0–47.6)^d^ 0.9 (0.2–4.6)^b^ 2.0 (0.2–16.7)^c^
		Wrist		3.3 (1.0–10.5)^i^				0.3 (0.0–5.9)	5.7 (1.8–18.0)^d^ 1.8 (0.7–4.4)^b^ 3.9 (1.2–12.2)^c^
		Hand		2.9 (1.0–8.4)^i^				0.5 (0.1–4.2)^h^	2.3 (0.6–8.6)^d^ 1.2 (0.5–2.7)^b^ 6.3 (2.4–16.4)^c^
Kok et al. (2013a, b)	12-month prevalence, not playing related	Total body	83 %		93 %		86 %		94 %^e^ 100 %^f^
	Point prevalence, not playing related		62 %		63 %		29 %		71 %^e^ 100 %^f^
Fishbein et al. (1988); Middlestadt and Fishbein (1988); Middlestadt and Fishbein (1989)	Point prevalence playing related	Total body	66 %		48 %		32 %		60 %^g^
Ackermann et al. (2012), Kenny and Ackermann (2015)	Point prevalence, playing related	Total body	Upper 45 % Lower 54 %		49 %		55 %		50 %^b^
Arnason et al. (2014)	Lifetime prevalence, playing related	Total body	67 %		Woodwinds and brass: 59 %	69 %^c^ 0 %^b^ *(n* = *3)*			
Steinmetz et al. (2015)	Lifetime prevalence, playing related	Total body	Upper: 90 % Lower: 91 %		87 %		84 %		85 %^b^

^a^OR compared to strings, unless otherwise stated

^b^Percussion

^c^Keyboard

^d^Guitar

^e^Percussion and keyboard

^f^Plucked strings

^g^Unspecified

^h^Horn

^i^Violin

### Age

One study compares lifetime prevalence rates of musculoskeletal complaints among age groups (Abreu-Ramos and Micheo 2007). The highest prevalence rates were reported in the highest (50–61 years; 91 %) and youngest (22–29, 83 %) age groups.

### Anatomic region

Above-mentioned differences in the reporting of complaints are also reflected in the heterogeneity of studied body areas. The number of reported body areas differed from four (e.g., neck, shoulder (both/left/right), fingers (each separate, or in general) up to 32. This high variability between affected anatomical areas (i.e., heterogeneity in location of complaints) made comparison between the included studies difficult, since multiple complaints at several anatomic regions can be present, as well as radiation of these complaints to different anatomical regions. In Table 7, the prevalence rates for each anatomic region are presented. Overall, the neck and shoulders were most frequently affected, and the elbows had the lowest prevalence rate of musculoskeletal complaints. No differences between left and right side of the body were evident.

**Table 7:** Prevalence rates of musculoskeletal complaints among professional musicians; anatomic region results

	Type of prevalence	Neck	Upper back	Lower back	Shoulders	Elbows	Wrists	Hands	Other
Leaver et al. (2011)	12-month prevalence, not playing related	56 %		51 %	51 %	21 %	Wrist + hands: 33 %		
	4-week prevalence, not playing related	36 %		33 %	37 %	12 %	Wrists + hands: 24 %		
Kaufman-Cohen and Ratzon (2011)	12-month prevalence, playing related	39 %	42 %	49 %	55 %				
Abreu-Ramos and Micheo (2007)									Only figure presented, no exact prevalence rates
Kaneko et al. (2005)	Point prevalence, not playing related	7 %	7 %	11 %	L: 7 % R: 6 %	L: 4 % R: 6 %	L: 6 % R: 5 %	L: 8 % R:5 %	
Engquist et al. (2004)	Point prevalence, not playing related	21 %	13 %	14 %	26 %	6 %		10 %	
	12-month prevalence, playing related	21 %	16 %	6 %	22 %	10 %		10 %	
	Chronic prevalence^a^	18 %	12 %	8 %	19 %	3 %		8 %	
Davies and Mangion (2002)									No information presented
Roach et al. (1994)	4-week prevalence, not playing related	40 %	28 %	26 %	28 %	6 %	14 %	20 %	Hips: 0 % Knees: 17 % Ankles/feet: 8 %
O’Neill et al. (2001)									(Among violinists) ‘of note, is the preponderance of problems in the neck and upper back, and the greater number of injuries on the left side of the neck where the violin is held’
Kok et al. (2013a, b)	12-month prevalence, not playing related	46 %	19 %	40 %	L: 28 % R: 30 %	L: 7 % R: 2 %	L: 16 % R: 17 %	L: 8 % R: 17 %	Knee L: 6 %; Knee R: 6 % Hip L: 4 %; Hip R: 4 % Ankle L: 4 %; Ankle R: 2 % Foot L: 6 %; Foot R: 2 % Jaw: 16 %
Fishbein et al. (1988); Middlestadt and Fishbein (1988); Middlestadt and Fishbein (1989)	Point prevalence, not playing related	L: 22 % R: 21 %	L: 16 % R: 16 %	L: 22 % R: 22 %	L: 20 % R: 20 %	L: 8 % R: 10 %	L: 9 % R: 10 %	L: 14 % R: 9 %	Finger L: 16 %; Finger R: 9 and Forearm L: 8 %; Forearm R: 7 % Middle back L: 11 %; Middle back R: 11 % Hip L: 3 %; Hip R: 3 % Knee L: 4 %; Knee R: 4 % Calf L: 1 %; Calf R: 1 % Ankle L: 2 %; Ankle R: 2 % Foot L: 2 %; Foot R: 2 % Toe L: 1 %; Toe R: 1 %
	Point prevalence playing related (=severe)	L: 12 % R: 13 %	L: 8 % R: 9 %	L: 11 % R: 13 %	L: 11 % R: 13 %	L: 4 % R: 6 %	L: 5 % R: 5 %	L: 10 % R: 5 %	Finger L: 9 %; Finger R: 5 and Forearm L: 5 %; Forearm R: 4 % Middle back L: 5 %; Middle back R: 5 % Hip L: 1 %; Hip R: 1 % Knee L: 1 %; Knee R: 1 % Calf L: 0 %; Calf R: 0 % Ankle L: 0 %; Ankle R: 0 % Foot L: 0 %; Foot R: 0 % Toe L: 0 %; Toe R: 0 %
Ackermann et al. (2012), Kenny and Ackermann (2015)	Point prevalence, playing related	14 %	12 %	8 %	Shoulder and upper arm L: 6 % Shoulder and upper arm R: 11 %	Elbow and forearm L:3 % Elbow and forearm R: 4 %	Wrist and hand L: 4 % Wrist and hand R: 3 %		
Steinmetz et al. (2015)	Lifetime prevalence, playing related	73 %	24 %	51 %	L: 55 % R: 52 %	L: 17 % R: 20 %	L: 55 % R: 24 %	Fingers L: 17 % Fingers R: 20 %	30 %^b^ 26 %^c^
	Point prevalence, playing related	18 %	7 %	9 %	L: 12 % R: 10 %	L: 4 % R: 4 %	L: 4 % R: 4 %	Fingers L: 5 % Fingers R: 2 %	4 %^b^ 3 %^c^
	3-month prevalence, playing related	30 %	11 %	22 %	L: 21 % R: 22 %	L: 6 % R: 6 %	L: 7 % R: 6 %	Fingers L: 8 % Fingers R: 5 %	9 %^b^ 7 %^c^

^a^Pain often, or all the time, in contrast to never, once or twice, sometimes during the last 12 months

^b^Teeth/jaw

^c^Tempomandibular joint

## Discussion

This systematic review focused on the prevalence of musculoskeletal complaints among professional musicians. In the included articles, there was a wide variability in the definition of these complaints as well as on the outcome measures used. The point prevalence of all musculoskeletal complaints ranged between 9 and 68 % and for playing-related musculoskeletal complaints between 9 and 68 %. Twelve-month prevalence ranged between 86 and 89 %, and playing-related 12-month prevalence ranged between 41 and 93 %. Playing-related lifetime prevalence ranged between 62 and 93 %. In most studies, women have a higher prevalence of complaints compared to men.

### Limitations of this study

Due to heterogeneity on several aspects between the studies in this systematic review, pooling of study data was not possible. Since the critical review of Zaza et al. (1998), more than a 100 new articles describing musculoskeletal complaints among musicians were published. Of these articles, 12 were included in this review. Many of these recently published articles lack essential methodological information (e.g. biased or non-described selection of participants, lack of reporting a response rate or a clear cut definition of the measured complaints). Also, the results section is often lacking important information (e.g. location as well as duration of the complaints). Furthermore, selection bias is often present in these studies. The latter is exemplified by missing general baseline information, like age and gender on the study subjects as well as which patients are selected to be included in the study and what the loss of follow-up is (i.e., response rate) (Eller et al. 1992; Fjellman-Wiklund and Chesky 2006; Kreutz et al. 2008; Hagglund and Jacobs 1996; Larsson et al. 1993; Miller et al. 2002; Brandfonbrener 1997; Nemoto and Arino 2007; Mehrparvar et al. 2012; Marques et al. 2003; Caldron et al. 1986; Arnason et al. 2014; de Sousa et al. 2014).

As described in the methods section, all articles lacking a clear description of the study population or a measured prevalence rate were excluded from this review. Therefore, the quality of the included studies in this review is generally high compared with the overall performing arts medicine literature. This is confirmed by the used methodological quality score on which 17 out of 21 studies score high.

Another limitation of this study is the lack of ‘non-classical’ professional musicians, e.g., musicians playing in a marching or pop/rock band. As these musicians have both another musculoskeletal load (e.g. standing performance instead of sitting) and another lifestyle, they possibly have other musculoskeletal problems compared to the classically trained musicians.

### Musculoskeletal complaints in musicians and subgroups at risk

We found that females have a higher prevalence of musculoskeletal complaints when compared with men, and this is in line with the literature of musculoskeletal complaints in the general population: female gender is a known risk factor for development of these complaints (Picavet and Schouten 2003).

Although comparison of the studies describing prevalence rates in music academy students and professional musicians was difficult due to heterogeneity, no evident difference in prevalence rate between music academy students and professional orchestra musicians was found.

Comparison of prevalence rates of musculoskeletal complaints between musicians who play different instruments did not show a specific instrumental group with an evidently highest prevalence rate, although brass instrument players had the lowest prevalence. It should be noted that some of the musicians play multiple instruments, where all included studies describe only the main instrument. Also, the instrument categories used consisted of instruments which are varying in size and playing position and technique. For example, the category strings consists of violin, viola (somewhat larger and heavier compared to the violin), cello and base players and in some studies even guitar players. The playing position of a base player is completely different compared with a violinist, and since the instrument is larger, a sitting position is used, and the repertoire of the base player (heavy, slow and often repetitive) is different compared with the fast and virtuoso repertoire of the violin. Thus, since the included articles combine the prevalence rates in groups of players, no distinction between subgroups of string players can be made.

No valid conclusion can be drawn from this review concerning the relation between age and musculoskeletal complaints among professional musicians. Only one study compared age groups, but this study used a lifetime prevalence rate, and the risk of recall bias is high when using lifetime prevalence rates (Moffitt et al. 2010). However, musculoskeletal complaints in the general population are most frequent among subjects in the fifth, sixth and seventh decade of their life (Picavet and Schouten 2003; Huisstede et al. 2008, 2006). As musicians pass through the same aging process, it is supposed that the highest prevalence of musculoskeletal complaints among them would be the same compared to the general population. However, there might be a ‘healthy player effect,’ in which musicians with severe musculoskeletal complaints quit their career before reaching this age. Therefore, musicians could have another distribution of musculoskeletal complaints in age groups compared to the general population.

### PRMDs/non-PRMDs

The term ‘PRMDs’ was introduced to evaluate musculoskeletal symptoms which interfere with the ability to play the instrument (Zaza et al. 1998). Since then, many studies evaluated these playing-related symptoms instead of evaluating all musculoskeletal symptoms, thereby excluding minor symptoms (Zaza and Farewell 1997; Davies and Mangion 2002; O’Neill et al. 2001; Ackermann et al. 2012). The use of this term has an important advantage; symptoms without impact on the musician (and therefore irrelevant symptoms) are excluded. However, the comparison of musicians with non-musicians is difficult with this definition. Besides, although Zaza et al. (1998) made a clear definition of the term PRMD, studies using other descriptions of the term are published (Davies and Mangion 2002; Abreu-Ramos and Micheo 2007). The current definition of PRMD does not include a causality of the complaints (i.e., is the complaint the result of playing of the instrument, or is it the result of a trauma and influences the complaint the ability to play the instrument).

### Recommendations for future research

We recommend that future research should aim for a higher level of methodological quality to contribute to the existing knowledge of the occurrence and risk factors for musicians’ musculoskeletal complaints. A minimum requirement is data on the included cohort, a brief definition of the measured musculoskeletal complaints (i.e. anatomic area, radiation etc.), data on loss of follow-up and the use of validated outcome measures. Focus should be on selecting subjects while avoiding bias (adequate response rate, describing non-responders and selection procedure), using adequate and validated instruments for measuring all outcomes; Using the DASH, SF-36, Michigan hand score, Nordic Questionnaire etc., has strong preference above using a non-validated self-made or adapted (modified existing, and not re-validated) questionnaire (Poolman et al. 2009). Scientific guidelines, for example the STROBE or IDEAL and NOS are recommended for increasing the quality of future studies (Elm et al. 2007; McCulloch et al. 2009).

## Conclusion

Musculoskeletal symptoms are highly prevalent among musicians, especially among women. In contrast to the literature on musculoskeletal complaints in the general population, evidence is scarce concerning prevalence rates in subgroups of age or occupation. Future research concerning the epidemiology of musculoskeletal complaints among musicians should focus on associated risk factors and follow the current guidelines (McCulloch et al. 2009; Elm et al. 2007) to optimize scientific quality.

## Appendix A: Literature search

(Prevalence OR prevalence* OR incidence OR incidence* OR morbidity OR morbidit* OR epidemiology OR epidemics OR frequency OR surveillance OR outbreaks OR endemics OR mortality OR occurrence) AND (“Musculoskeletal Diseases”[Mesh] OR “Musculoskeletal System”[Mesh] OR “Musculoskeletal Physiological Phenomena”[Mesh] OR musculoskeletal OR musculo-skeletal OR “musculo skeletal” OR “musculoskeletal complaints” OR “musculoskeletal complaint” OR “musculoskeletal problems” OR “musculoskeletal problem” OR “musculoskeletal disorders” OR “musculoskeletal disorder” OR “Musculoskeletal Diseases” OR bone OR bones OR skeletal OR skeleton OR tendon OR tendons OR (joint NOT “joint improvisation”) OR joints OR arthritis OR osteoarthritis OR shoulder OR shoulders OR wrist OR wrists OR knee OR knees OR hip OR hips OR elbow OR elbows OR leg OR legs OR arm OR hand OR hands OR feet OR foot OR spine OR spinal OR disc OR discs OR disk OR disks OR neck OR extremity OR extremities OR feet OR foot OR “Face”[Mesh] OR “face”[tw] OR orofacial*[tw] OR “facial”[tw] OR “Facial Pain”[Mesh] OR “Facial Nerve Diseases”[Mesh] OR “Facial Muscles”[Mesh] OR “Cheek”[tw] OR “Chin”[tw] OR “Eye”[tw] OR “Eyebrows”[tw] OR “Cheeks”[tw] OR “Chins”[tw] OR “Eyes”[tw] OR “Eyebrow”[tw] OR “Eyelids”[tw] OR “Eyelid”[tw] OR “Conjunctiva”[tw] OR “Eyelashes”[tw] OR “Eyelash”[tw] OR “Meibomian Glands”[tw] OR “Meibomian Gland”[tw] OR “Forehead”[tw] OR “Mouth”[tw] OR “Lip”[tw] OR “Nasolabial Fold”[tw] OR “Nose”[tw] OR “Foreheads”[tw] OR “Mouths”[tw] OR “Lips”[tw] OR “Nasolabial Folds”[tw] OR “Noses”[tw] OR “Parotid Region”[tw] OR “Nasal”[tw] OR “Facial Injuries”[Mesh] OR PRMDs OR PRMD OR muscle OR muscles OR myopathy OR myopathies OR dystonia) AND (((music OR “Music”[mesh]) AND (“Occupational Diseases”[mesh] OR occupation OR occupational OR occupation*)) OR musicians OR musician OR musician* OR “musical performance” OR “music academy students” OR “music students” OR “instrument players” OR “instrument player” OR pianist OR pianists OR “piano playing” OR “piano player” OR “piano players” OR violinist OR violinists OR “violin player” OR “violin players” OR “viola player” OR “viola players” OR cellist OR cellists OR “cello player” OR “cello players” OR “double base player” OR “double base players” OR “bass player” OR “bass players” OR bassist OR bassists OR “flute player” OR “flute players” OR flutist OR flutists OR “oboe player” OR “oboe players” OR oboeist OR oboeists OR “clarinet player” OR “clarinet players” OR clarinetist OR clarinetists OR “bassoon player” OR “bassoon players” OR bassoonist OR bassoonists OR “trumpet player” OR “trumpet players” OR trumpetist OR trumpetists OR trumpeter OR trumpeters OR “trombone player” OR “trombone players” OR trombonist OR trombonists OR “tuba player” OR “tuba players” OR “horn player” OR “horn players” OR hornist OR hornists OR “percussion player” OR “percussion players” OR percussionist OR percussionists OR “harp player” OR “harp players” OR harpist OR harpists OR “organ player” OR “organ players” OR organist OR organists OR “guitar player” OR “guitar players” OR guitarist OR guitarists OR “string player” OR “string players” OR “woodwind player” OR “woodwind players” OR “wind instrument player” OR “wind instrument players” OR “brass players” OR “brass player” OR drummer OR drummers OR “piano playing” OR “violin playing” OR “viola playing” OR “cello playing” OR “double base playing” OR “bass playing” OR “flute playing” OR “oboe playing” OR “clarinet playing” OR “bassoon playing” OR “trumpet playing” OR “trombone playing” OR “tuba playing” OR “horn playing” OR “percussion playing” OR “harp playing” OR “organ playing” OR “guitar playing” OR “string playing” OR “woodwind playing” OR “wind instrument playing” OR “brass playing”).

## References

[CR1] Abreu-Ramos AM, Micheo WF (2007). Lifetime prevalence of upper-body musculoskeletal problems in a professional-level symphony orchestra: age, gender, and instrument-specific results. MPPA.

[CR2] Ackermann B, Driscoll T, Kenny DT (2012). Musculoskeletal pain and injury in professional orchestral musicians in Australia. MPPA.

[CR3] Arnason K, Arnason A, Briem K (2014). Playing-related musculoskeletal disorders among icelandic music students: differences between students playing classical vs rhythmic music. MPPA.

[CR4] Barton R, Kallian C, Bushee M, Callen J, Cupp T, Ochs B, Sharp M, Tetrault K (2008). Occupational performance issues and predictors of dysfunction in college instrumentalists. MPPA.

[CR5] Bejjani FJ, Kaye GM, Benham M (1996). Musculoskeletal and neuromuscular conditions of instrumental musicians. Arch Phys Med Rehabil.

[CR6] Brandfonbrener AG (1997). Orchestral injury prevention intervention study. Med Probl Perform Artist.

[CR7] Caldron PH, Calabrese LH, Clough JD, Lederman RJ, Williams G, Leatherman J (1986). A survey of musculoskeletal problems encountered in high-level musicians. MPPA.

[CR8] Chimenti RL, Van Dillen LR, Prather H, Hunt D, Chimenti PC, Khoo-Summers L (2013). Underutilization of worker's compensation insurance among professional orchestral musicians. Med Probl Perform Art..

[CR9] Chesky K (2000). UNT-MHS website.

[CR10] Davies J, Mangion S (2002). Predictors of pain and other musculoskeletal symptoms among professional instrumental musicians: elucidating specific effects. MPPA.

[CR11] de Sousa CMG, Greten HJ, Machado J, Coimbra D (2014). The Prevalence of Playing-related Musculoskeletal Disorders (PRMSD) Among Professional Orchestra players. Musica Hodie.

[CR12] Eller N, Skylv G, Ostri B, Dahlin E, Suadicani P, Gyntelberg F (1992). Health and lifestyle characteristics of professional singers and instrumentalists. Occup Med (Lond).

[CR13] von Elm E, Altman DG, Egger M, Pocock SJ, Gotzsche PC, Vandenbroucke JP (2007). The Strengthening the Reporting of Observational Studies in Epidemiology (STROBE) statement: guidelines for reporting observational studies. Lancet.

[CR14] Engquist K, Orbaek P, Jakobsson K (2004). Musculoskeletal pain and impact on performance in orchestra musicians and actors. MPPA.

[CR15] Fishbein M, Middlestadt SE, Ottati V, Straus S, Ellis A (1988). Medical problems among ICSOM musicians: overview of a national survey. MPPA.

[CR16] Fjellman-Wiklund A, Chesky K (2006). Musculoskeletal and general health problems of acoustic guitar, electric guitar, electric bass, and banjo players. Med Probl Perform Artist.

[CR17] Fotiadis DG, Fotiadou EG, Kokaridas DG, Mylonas AC (2013). Prevalence of musculoskeletal disorders in professional symphony orchestra musicians in Greece: a pilot study concerning age, gender, and instrument-specific results. MPPA.

[CR18] Guptill C, Golem MB (2008). Case study: musicians’ playing-related injuries. Work.

[CR19] Hagglund KL, Jacobs K (1996). Physical and mental practices of music students as they relate to the occurrence of music-related injuries. Work.

[CR20] Heinan M (2008). A review of the unique injuries sustained by musicians. JAAPA.

[CR21] Hoppmann RA, Reid RR (1995). Musculoskeletal problems of performing artists. Curr Opin Rheumatol.

[CR22] Huisstede BM, Bierma-Zeinstra SM, Koes BW, Verhaar JA (2006). Incidence and prevalence of upper-extremity musculoskeletal disorders. A systematic appraisal of the literature. BMC Musculoskelet Disord.

[CR23] Huisstede BM, Wijnhoven HA, Bierma-Zeinstra SM, Koes BW, Verhaar JA, Picavet S (2008). Prevalence and characteristics of complaints of the arm, neck, and/or shoulder (CANS) in the open population. Clin J Pain.

[CR24] Kaneko Y, Lianza S, Dawson WJ (2005). Pain as an incapacitating factor in symphony orchestra musicians in Sao Paulo. Brazil. MPPA.

[CR25] Kaufman-Cohen Y, Ratzon NZ (2011). Correlation between risk factors and musculoskeletal disorders among classical musicians. Occup Med (Lond).

[CR26] Kenny D, Ackermann B (2015). Performance-related musculoskeletal pain, depression and music performance anxiety in professional orchestral musicians: a population study. Psychol Music.

[CR27] Kok LM, Vlieland TP, Fiocco M, Nelissen RG (2013). A comparative study on the prevalence of musculoskeletal complaints among musicians and non-musicians. BMC Musculoskelet Disord.

[CR28] Kok LM, Vliet Vlieland TP, Fiocco M, Kaptein AA, Nelissen RG (2013). Musicians’ illness perceptions of musculoskeletal complaints. Clin Rheumatol.

[CR29] Kreutz G, Ginsborg J, Williamon A (2008). Music students’ health problems and health-promoting behaviours. MPPA.

[CR30] Kuorinka I, Jonsson B, Kilbom A, Vinterberg H, Biering-Sorensen F, Andersson G, Jorgensen K (1987). Standardised Nordic questionnaires for the analysis of musculoskeletal symptoms. Appl Ergon.

[CR31] Larsson LG, Baum J, Mudholkar GS, Srivastava DK (1993). Hypermobility: prevalence and features in a Swedish population. Br J Rheumatol.

[CR32] Leaver R, Harris EC, Palmer KT (2011). Musculoskeletal pain in elite professional musicians from British symphony orchestras. Occup Med (Lond).

[CR33] Loney PL, Chambers LW, Bennett KJ, Roberts JG, Stratford PW (1998). Critical appraisal of the health research literature: prevalence or incidence of a health problem. Chronic Dis Can.

[CR34] Lopez TM, Farias MJ (2013). Strategies to promote health and prevent musculoskeletal injuries in students from the high conservatory of music of Salamanca, Spain. MPPA.

[CR35] Marques DN, Rosset-Llobet J, Marques F, Gurgel IGD, Augusto LGS (2003). Flamenco guitar as a risk factor for overuse syndrome. Med Probl Perform Artist.

[CR36] McCulloch P, Altman DG, Campbell WB, Flum DR, Glasziou P, Marshall JC, Nicholl J, Aronson JK, Barkun JS, Blazeby JM, Boutron IC, Campbell WB, Clavien PA, Cook JA, Ergina PL, Feldman LS, Flum DR, Maddern GJ, Nicholl J, Reeves BC, Seiler CM, Strasberg SM, Meakins JL, Ashby D, Black N, Bunker J, Burton M, Campbell M, Chalkidou K, Chalmers I, de Leval M, Deeks J, Ergina PL, Grant A, Gray M, Greenhalgh R, Jenicek M, Kehoe S, Lilford R, Littlejohns P, Loke Y, Madhock R, McPherson K, Meakins J, Rothwell P, Summerskill B, Taggart D, Tekkis P, Thompson M, Treasure T, Trohler U, Vandenbroucke J (2009). No surgical innovation without evaluation: the IDEAL recommendations. Lancet.

[CR37] Mehrparvar AH, Mostaghaci M, Gerami RF (2012). Musculoskeletal disorders among Iranian instrumentalists. Med Probl Perform Art.

[CR38] Middlestadt SE, Fishbein M (1988). Health and occupational correlates of perceived occupational stress in symphony orchestra musicians. J Occup Med.

[CR39] Middlestadt SE, Fishbein M (1989). The Prevalence of Severe Musculoskeletal Problems Among Male and Female Symphony-Orchestra String Players. MPPA.

[CR40] Miller G, Peck F, Watson JS (2002). Pain disorders and variations in upper limb morphology in music students. Med Probl Perform Artist.

[CR41] Mishra W, De A, Gangopadhyay S, Chandra AM (2013). Playing-related musculoskeletal disorders among Indian tabla players. MPPA.

[CR42] Moffitt TE, Caspi A, Taylor A, Kokaua J, Milne BJ, Polanczyk G, Poulton R (2010). How common are common mental disorders? Evidence that lifetime prevalence rates are doubled by prospective versus retrospective ascertainment. Psychol Med.

[CR43] Nemoto K, Arino H (2007). Hand and upper extremity problems in wind instrument players in military bands. Med Probl Perform Artist.

[CR44] O’Neill L, Taunton J, MacIntyre DL (2001). Making music: challenging the physical limits of the human body: a survey of musicians in western Canada. Physiother Can.

[CR45] Paarup HM, Baelum J, Holm JW, Manniche C, Wedderkopp N (2011). Prevalence and consequences of musculoskeletal symptoms in symphony orchestra musicians vary by gender: a cross-sectional study. BMC Musculoskelet Disord.

[CR46] Parry CB (2003). Prevention of musicians’ hand problems. Hand Clin.

[CR47] Picavet HS, Schouten JS (2003). Musculoskeletal pain in the Netherlands: prevalences, consequences and risk groups, the DMC(3)-study. Pain.

[CR48] Poolman RW, Swiontkowski MF, Fairbank JC, Schemitsch EH, Sprague S, de Vet HC (2009). Outcome instruments: rationale for their use. J Bone Joint Surg Am.

[CR49] Roach KE, Martinez MA, Anderson N (1994). Musculoskeletal Pain in Student Instrumentalists—A Comparison with the General Student Population. MPPA.

[CR50] Sataloff RT, Brandfonbrener AG, Lederman RJ (2010). Performing arts medicine.

[CR51] Shamliyan T, Kane RL, Dickinson S (2010). A systematic review of tools used to assess the quality of observational studies that examine incidence or prevalence and risk factors for diseases. J Clin Epidemiol.

[CR52] Shields N, Dockrell S (2000). The prevalence of injuries among pianists in music schools in Ireland. Med Probl Perform Artist.

[CR53] Silva AG, La FM, Afreixo V (2015). Pain prevalence in instrumental musicians: a systematic review. MPPA.

[CR54] Spahn C, Hildebrandt H, Seidenglanz K (2001). Effectiveness of a prophylactic course to prevent playing-related health problems of music students. MPPA.

[CR55] Steinmetz A, Scheffer I, Esmer E, Delank KS, Peroz I (2015). Frequency, severity and predictors of playing-related musculoskeletal pain in professional orchestral musicians in Germany. Clin Rheumatol.

[CR56] Wood GC (2014). Prevalence, risk factors, and effects of performance-related medical disorders (PRMD) among tertiary-trained jazz pianists in Australia and the United States. MPPA.

[CR57] Wristen BG, Fountain SE (2013). Relationships Between Depression, Anxiety, and Pain in a Group of University Music Students. MPPA.

[CR58] Zaza C (1998). Playing-related musculoskeletal disorders in musicians: a systematic review of incidence and prevalence. CMAJ.

[CR59] Zaza C, Farewell VT (1997). Musicians’ playing-related musculoskeletal disorders: an examination of risk factors. Am J Ind Med.

[CR60] Zaza C, Charles C, Muszynski A (1998). The meaning of playing-related musculoskeletal disorders to classical musicians. Soc Sci Med.

[CR61] Zetterberg C, Backlund H, Karkson J, Werner H, Olsson L (1998). Musculoskeletal problems among male and female music students. MPPA.

